# A Large Animal Survival Model to Evaluate Bariatric Surgery Mechanisms

**DOI:** 10.4236/ss.2015.68050

**Published:** 2015-07-24

**Authors:** Vlad V. Simianu, Jonathan G. Sham, Andrew S. Wright, Skye D. Stewart, Mouhamad Alloosh, Michael Sturek, David E. Cummings, David R. Flum

**Affiliations:** 1 Departments of Surgery, University of Washington, Seattle, USA; 2 Department of Cellular and Integrative Physiology, Indiana University School of Medicine, Indianapolis, USA; 3 Departments of Medicine, University of Washington, Seattle, USA; 4 Departments of Health Services, University of Washington, Seattle, USA

**Keywords:** Ossabaw, Bariatric Surgery, Roux-en-Y Gastric Bypass, Weight Loss, Metabolic Syndrome

## Abstract

**Background:**

The impact of Roux-en-Y gastric bypass (RYGB) on type 2 diabetes mellitus is thought to result from upper and/or lower gut hormone alterations. Evidence supporting these mechanisms is incomplete, in part because of limitations in relevant bariatric-surgery animal models, specifically the lack of naturally insulin-resistant large animals. With overfeeding, Ossabaw swine develop a robust metabolic syndrome, and may be suitable for studying post-surgical physiology. Whether bariatric surgery is feasible in these animals with acceptable survival is unknown.

**Methods:**

Thirty-two Ossabaws were fed a high-fat, high-cholesterol diet to induce obesity and insulin resistance. These animals were assigned to RYGB (n = 8), RYGB with vagotomy (RYGB-V, n = 5), gastrojejunostomy (GJ, n = 10), GJ with duodenal exclusion (GJD, n = 7), or sham operation (n = 2) and were euthanized 60 days post-operatively. Post-operative changes in weight and food intake are reported.

**Results:**

Survival to scheduled necropsy among surgical groups was 77%, living an average of 57 days post-operatively. Cardiac arrest under anesthesia occurred in 4 pigs. Greatest weight loss (18.0% ± 6%) and food intake decrease (57.0% ± 20%) occurred following RYGB while animals undergoing RYGB-V showed only 6.6% ± 3% weight loss despite 50.8% ± 25% food intake decrease. GJ (12.7% ± 4%) and GJD (1.2% ± 1%) pigs gained weight, but less than sham controls (13.4% ± 10%).

**Conclusions:**

A survival model of metabolic surgical procedures is feasible, leads to significant weight loss, and provides the opportunity to evaluate new interventions and subtle variations in surgical technique (e.g. vagus nerve sparing) that may provide new mechanistic insights.

## 1. Introduction

Roux-en-Y gastric bypass surgery (RYGB) promotes substantial, sustained weight loss [1] [2], and it is the most effective method to ameliorate obesity-related comorbidity including type 2 diabetes mellitus (T2DM) [3]-[5]. Because major improvements in T2DM typically occur prior and out of proportion to significant body weight loss [6], the impact of RYGB on T2DM may not result from weight loss and reduced caloric intake alone. Important unanswered questions regarding the anti-diabetes effects of RYGB remain, including contributions of the proximal vs. distal intestines in T2DM remission, the reversibility of improved glycemic control with foregut exposure to food, and roles of the vagus nerve in these effects.

Over the past several years, our group has developed a porcine survival model for metabolic surgery [7] and more recently extended that work to address one of the major shortcomings of large animals as models for metabolic surgery. Over many generations, animal husbandry practices have limited the gene pool of large animals such that there are no natural models of “unhealthy” obesity. Farm animals rendered obese typically do not develop insulin resistance or heart disease, and consequently, large animal models of metabolic disease have been limited [8]. Classically, larger animals are given toxins (e.g. streptozotocin) to impair pancreatic function [9], while small animal models depend on knockout or gene silencing techniques to mimic human insulin resistance.

Although a naturally occurring large animal model of diabetes does not exist, in the 1970s, on Ossabaw Island near Georgia, a colony of pigs was discovered that exhibited many features useful for the study of bariatric surgery. Abundant natural resources exist on the island only seasonally, followed by periods of food scarcity. Through generations of natural selection, surviving Ossabaw pigs gain large amounts of weight during times of plenty, allowing them to survive seasonal famines. Exhibiting “thrifty genome” characteristics when provided unrestricted access to high-calorie diets [10]-[12], they develop obesity, insulin resistance and glucose intolerance [13], dyslipidemia [14] [15], and hypertension [14] [16], which are widely accepted characteristics of metabolic syndrome (MetS) [16]-[18].

The objective of this study was to determine the feasibility and appropriateness of bariatric surgical techniques in the Ossabaw survival model. The advantages of a large-animal model are its similarities with humans regarding techniques and anatomic features. More so than rodents, the upper GI tract is very similar in humans and pigs [19], and allows application of nearly identical surgical techniques and instruments. In obese Ossabaw pigs, we describe our initial experience with long-term survival surgery including RYGB without vagotomy, RYGB with vagotomy (RYGB-V), gastrojejunostomy with duodenal exclusion (GJD, which creates a gastric-sparing bypass of the segment of proximal intestine excluded in RYGB), and gastrojejunostomy (GJ, which is identical to the latter operation with duodenal inclusion). These variations were selected to better elucidate what had been dubbed the upper and lower intestinal hypotheses for endocrine/metabolic pathways of diabetes development and resolution.

## 2. Materials and Methods

All experimental procedures involving animals were approved by the Institutional Animal Care and Use Committee at the University of Washington (UW) with the recommendations outlined by the National Research Council and the American Veterinary Medical Association Panel on Euthanasia [20] [21].

### 2.1. Animals and Environment

Since January 2010, 32 female Ossabaw swine have entered the study protocol. Pigs were obtained from the joint Indiana University School of Medicine (IUSM) and Purdue University Facility. This study excludes 1 pig which died at IUSM before shipment. To promote weight gain and insulin resistance, animals were maintained on excess calorie high-fat, high-cholesterol diet (*vide infra*) for ≥180 days before arriving at UW, at age 12 - 18 months. Pigs were acclimated to the UW research facility for at least 7 days before undergoing initial vascular access catheter placement. Animals lived in a 70°F temperature-controlled room on a 12:12-h light:dark cycle. They had free access to drinking water and were removed at least once daily for stall cleaning. Experimental pigs were fed twice daily at fixed times a customized obesogenic TestDiet^®^ containing high levels of fructose, lipids, and cholesterol, with 16.1% proteins, 43.1% lipids, and 40.8% carbohydrates (3580 kcal/meal or 7160 kcal/day).

Twenty Ossabaw were fed standard, non-fat chow (~3000 kcal/day) and served as non-surgical, lean controls. Food intake was recorded daily. Change in food intake was derived from food volume left uneaten, measured at daily feeding times, and calculated as difference from preoperative baseline.

### 2.2. Intravascular Catheters

All animals were surgically implanted with vascular-access catheters to permit collection of serial blood samples and for medication administration (e.g., analgesics, antibiotics if necessary) postoperatively. Porcine animal models pose a special challenge for long-term vascular access given their size and lack of physical restraint [22]-[24]. Catheters were initially placed in the external jugular vein percutaneously, but early problems with this technique led us to switch to tunneled placement in the internal jugular vein under anesthesia. All tunneled placements used 12 Fr. Dual lumen Hickman Catheter (Bard Peripheral Vascular, Tempe, AZ) using a technique that has previously been described [25] [26]. Catheter replacements were required whenever catheters malfunctioned. Early in the study period, catheters were removed at 2 weeks post-operatively and replaced close to 60 days post-operatively, prior to necropsy. Following several anesthesia-related deaths, however, pigs in the later part of the study maintained catheters throughout the entire experimental period. Catheters were flushed at least twice daily with a heparin-saline solution then locked with a solution containing vancomycin (1.0 mg/ml).

### 2.3. Surgical Intervention

Pigs were randomly assigned to one of four GI operations (RYGB, RYGB-V, GJ, GJD) or a sham operation. All operations were standardized, and our pre-operative and anesthesia protocols have been previously reported [7]. For RYGB, a gastric pouch approximately 3 × 3 cm was created. The small bowel reconstruction approximated the human RYGB with approximately ~45 cm of biliary-pancreatic-duodenal (BPD) limb. However, given recognized variation in the intestinal length of Ossabawswine [19], the entire small bowel length was measured in each animal and one third of the small bowel used for an antecolic, antegastric alimentary limb. The RYGB-V is identical, but includes division of anterior and posterior vagus nerves as they cross the gastro-esophageal junction. Resection of nervous tissue was confirmed by histologic assessment. The GJ operation creates a connection between the mid-stomach and jejunum (as with RYGB procedures approximately one third of the distance on the small bowel), with full preservation of the stomach and pylorus. The GJD operation is similar to GJ, with additional division and detachment of the pylorus from the proximal duodenum, bypassing the same length of small bowel as in RYGB and RYGB-V. Sham surgery involves a full midline incision and bowel manipulation for ~130 minutes, the time of an average RYGB.

Early in the study, gastro-enteric anastomoses were performed using 4.5-mm GIA (United States Surgical, Norwalk, CT) stapler around the anvil of a 25-mm circular stapler. However, the thickened esophageal and gastric walls resulted in early anastomotic dehiscence at the esophageal-gastric junction, resulting in mediastinitis and/or peritonitis, and ultimately in untimely death in 5 animals. After these complications, we revised our protocol to hand-sewing for the gastric-jejunostomy anastomoses, and we created better practices for monitoring post-surgical outcomes to identify dehiscence within the first few post-operative days, using endoscopic exploration when animals began to deteriorate clinically. We continued to perform the side-to-side jejuno-jejunosto-my using a GIA stapler and 3-0 Maxon (United States Surgical, Norwalk, CT) reinforcement suture as we have previously described [7].

### 2.4. Statistical Analyses

Data analyses were descriptive rather than comparative, given the small number of animals involved. Pre-operative weights are reported as means with standard deviations (SD). Weight change is reported as a percentage change (±SD) using difference between pre-operative and pre-necropsy weights. Food intake change is reported as a percentage change (±SD) using average daily intake before and serially after surgery. Excel (version 12.3.6, Microsoft) was used for statistical analysis.

## 3. Results

Thirty-two Ossabaw swine have been studied to date, and survival and detailed perioperative complications are reported in Table 1. Six out of 8 (75%) pigs assigned to RYGB survived to scheduled necropsy. One that died post-operatively was euthanized on POD 35 after evisceration; necropsy revealed intra-abdominal adhesions but an intact anastomosis. The other died on POD 63, with necropsy showing severe necro-hemorrhagic enteritis and typhlocolitis, presumably from *Clostridium difficile* infection. Five pigs were assigned to RYGB-V. One had cardiac arrest during surgery and was not counted towards the overall survival. Two died before scheduled necropsy. One was re-explored on POD7 for anastomotic leak, and died during surgery. The other underwent revision of a laparotomy closure on POD2 and was subsequently euthanized on POD 11 from RYGB-V due to failure to thrive and PO intolerance. Necropsy revealed an intact but edematous gastro-jejunostomy.

Ten pigs were assigned to GJ surgery, with 8 of the 9 (89%) who underwent the operation surviving to scheduled necropsy. One pig died in the recovery phase of initial catheter placement and never underwent GJ, and was not counted towards the overall survival of the group. Another went into cardiac arrest during tunneled catheter replacement on POD 57; necropsy revealed pulmonary changes consistent with cardiac arrest. Seven pigs underwent GJD, and 6 (86%) survived to scheduled necropsy. One died on POD5; necropsy revealed pancreatitis. Two pigs underwent sham surgery, with only one surviving to scheduled necropsy. The other died on POD 75 from cardiac arrest during attempted tunneled catheter replacement.

**Figure 1:**
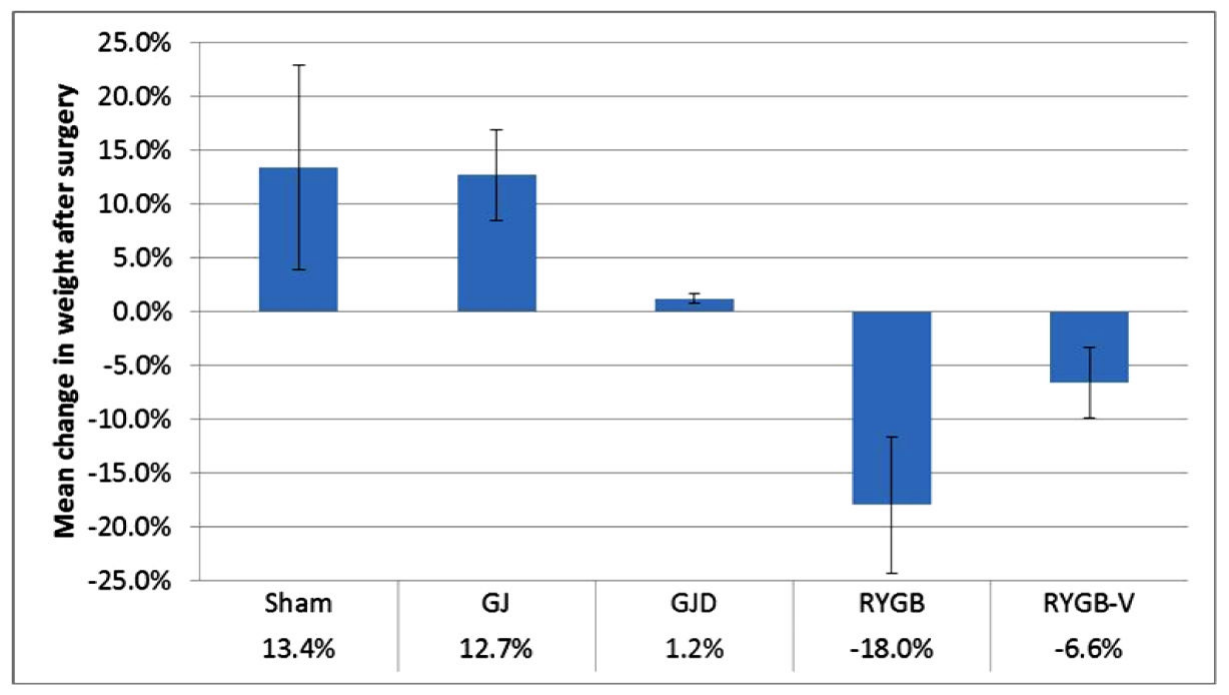
Weight change following surgery for Obesogenic-diet Ossabaws^a,b^. ^a^Mean change in weight reported as percentage (±SD) change from preoperative weigh (Sham: 78.1 ± 15.2 kg; GJ: 72.3 ± 8.0 kg; GJD: 79.8 ±8.1 kg; RYGB: 68.5 ± 7.0 kg; RYGB-V: 70.7 ± 6.7 kg); ^b^Only pigs surviving beyond immediate postoperative period (POD 35+) included in estimates (n_Sham_ = 2; n_GJ_ = 9; n_GJD_ = 6; n_RYGB_ = 8; n_RYGB-V_ = 2).

**Figure 2:**
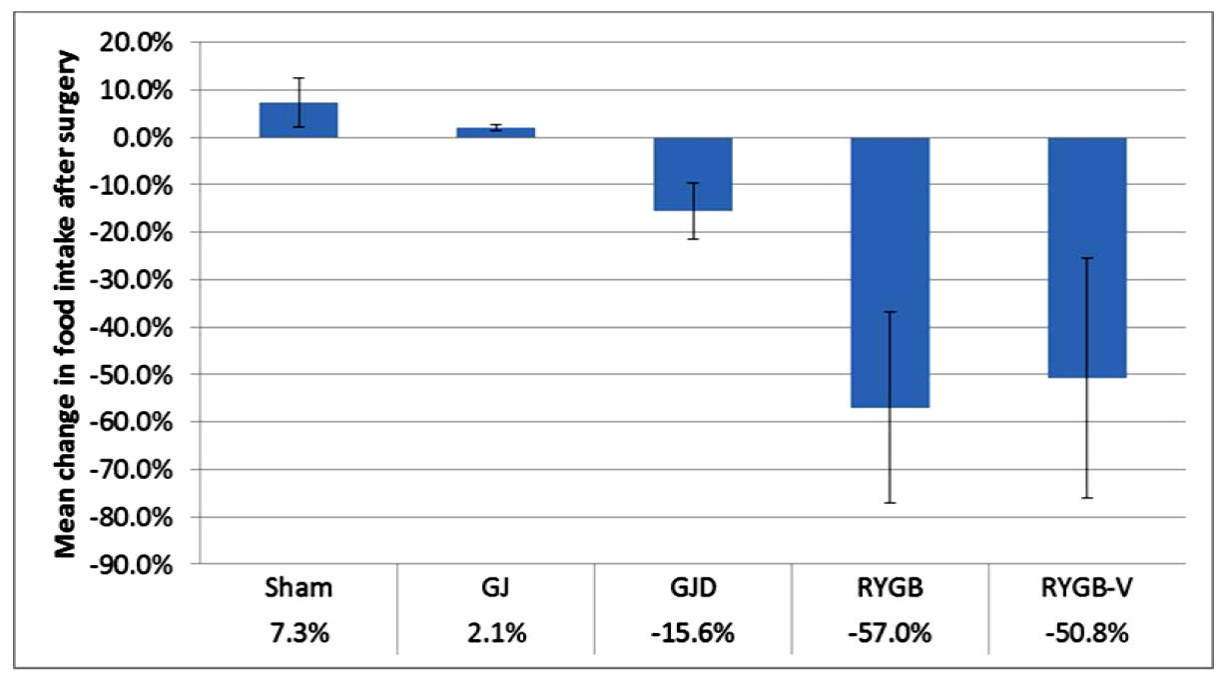
Change in food intake following surgery for Obese Ossabaws^a^. ^a^Only pigs surviving beyond immediate postoperative period (POD 35+) included in estimates (n_Sham_ = 2; n_GJ_ = 9; n_GJD_ = 6; n_RYGB_ = 8; n_RYGB-V_ = 2).

The mean preoperative weight of high-fat-fed Ossabaws was 73.4 ± 8.6 kg compared to 63.2 ± 12.9 kg in chow-fed Ossabaws. Preoperative weights for each surgical procedure were: Sham 78.1 ± 15.2 kg; GJ 72.3 ± 8.0 kg; GJD 79.8 ± 8.1 kg; RYGB 68.5 ± 7.0 kg; and RYGB-V 70.7 ± 6.7 kg. Postoperative weight and food intake change, stratified by operation, are shown in Figure 1 and Figure 2, respectively. Ossabaws undergoing sham operation gained 13.4% ± 9.5% weight and increased their food intake by 7.3% ± 5.2% over the experimental period. Over an equivalent period, GJ pigs gained 12.7% ± 4.2% weight and increased food intake by 2.1% ± 0.7%. Pigs in the GJD group gained 1.2% ± 0.5% weight but decreased their food intake by 15.6% ± 5.9%. Pigs in the RYGB and RYGB-V groups decreased their weight by 18.0% ± 6.4% and 6.6% ± 3.3% respectively, and decreased their food intake by 57.0% ± 20.2% and 50.8% ± 25.4%.

## 4. Discussion

Surgical approaches to obesity consistently promote major, sustained weight loss, and improvement or remission of many, obesity-related comorbidities. This reduction in comorbidities, most notably T2DM, cannot be explained solely by the effects of weight loss and reduced caloric intake, and there are several hypotheses regarding how GI rearrangement ameliorates diabetes [6] [27]. The “upper intestinal hypothesis” postulates that exclusion of the proximal small bowel from ingested nutrients exerts direct anti-diabetes effects, potentially through incompletely identified, nutrient-regulated factors or processes that influence glucose homeostasis [28] [29]. The “lower intestinal hypothesis” postulates that enhanced delivery of nutrients to the distal bowel augments glucagon-like peptide-1 secretion, increasing insulin secretion [30] [31]. In addition, compromised secretion of the pro-diabetic hormone ghrelin after RYGB might help improve glucose tolerance [32] [33]. Unfortunately, surgical experimental GI manipulations to test these hypotheses are often not practical in humans [34].

Rodents are the dominant model for metabolic surgical evaluations [19], but concerns over anatomic differences and variation in physiologic responses compared with humans limit their utility in studying feasibility of surgical techniques [35]. Insulin resistance in obese, large animals amenable to routine biomedical study has heretofore been lacking. Although swine appear to be a more appropriate model to study bariatric surgical changes because of their anatomic and functional GI similarity to humans [36] [37], as well as their equivalent hormonal responses to starvation and feeding [38]-[40], the lack of a swine model with insulin resistance has been problematic [8].

The Ossabaw miniature swine appears to have a form of naturally occurring, polygenic insulin resistance, along with a unique propensity to obesity [10]-[13] and dyslipidemia [14] [15]. Comparisons of Ossabaw swine to the well-established Yucatan swine model revealed that Ossabaws show greater propensity to obesity, especially visceral, than Yucatans on rigorously controlled experimental diets [14]-[17]. The miniature stature of Ossabaw pigs (50 - 70 kg at six months), coupled with metabolic-syndrome characteristics at this age, make them an ideal animal model for metabolic biomedical research. Having performed RYGB in other swine breeds [7], we hypothesized that the Ossabaw would be a superior model for studies of metabolic surgery and glucose homeostasis. Our investigations confirm that high-fat-fed Ossabaws had higher pre-operative weights compared to lean Ossabaws. In addition, evidence of increased levels of fasting glucose, insulin, and insulin resistance in these animals and the impact of the GI surgeries on glucose homeostasis, have been separately reported by our group [41].

The surgical survival model we developed is feasible and effective, but required trial and error manipulations, especially related to repeated anesthesia exposure. Although not all animals survived to necropsy, an important marker of a successful model is the ability to reproducibly perform “human-like” procedures and techniques and sustain enough animals to target survival dates. Three deaths occurred late in the study period from non-surgical causes, approximately at or beyond 60 days. We achieved 77% survival to scheduled necropsy, with intra-operative cardiac arrest being the leading cause of premature death. Three of the animals died during catheter placements, with two deaths from catheter replacement prior to scheduled necropsy. Two animals required re-exploration, and one died of intra-operative hemorrhage. These anesthesia complications were early in the model development (first 10 cases) and prompted important changes in our protocol to minimize anesthesia exposure. Only 3 unplanned deaths occurred during the second 22 cases (86% survival).

Our GI operations were designed to clarify mechanisms involved in bariatric post-surgical changes. As expected, our sham operation served as a control and did not lead to a decrease in weight or food intake. The GJ operation creates a moderate shortcut for food to pass from the intact stomach into the proximal jejunum, equivalent to the intestinal shortcut in our porcine RYGB but without gastric restriction or proximal intestinal bypass. By expediting delivery of ingested nutrients to the distal bowel, the operation discretely engages physiologic phenomena described in the lower intestinal hypothesis, but only to a degree of RYGB. In our study, the GJ did not inhibit weight gain, and the pigs had only a slight increase in food intake. These animals could serve in future, more sophisticated studies of glucose homeostasis to explore the distal intestinal hypothesis without confounding from weight loss.

The GJD procedure creates the same shortcut from stomach to jejunum as does GJ and RYGB, but unlike GJ, it excludes proximal intestinal nutrient exposure similar to a traditional RYGB. Comparing the effects of GJD vs. GJ on glucose homeostasis will allow us to distinguish discrete consequences of phenomena described in the upper and lower intestinal hypotheses. Neither of these procedures is confounded by any added gastric alterations, nor do they cause weight loss in our pigs. We have previously reported how these operations should highlight the weight-independent effects of distal intestinal nutrient exposure vs. proximal intestinal nutrient exclusion on glucose homeostasis [41], and studies describing hormonal alterations in these pigs are underway by our group.

Along with the upper and lower intestinal pathways influenced by the GJ and GJD operations, RYGB reduces gastric capacity. By comparing this operation with the other two, we can discriminate effects on glucose homeostasis that are related to gastric change and/or weight loss from those that result purely from the upper and lower intestinal hypotheses. The role of the vagus nerve in glucose homeostasis can be studied with the RYGB-V operation. The GI tract is heavily innervated by the vagus, which mediates many effects of gut peptides on satiety and glucose homeostasis [42] [43]. Hence, vagotomy might be expected to promote increased meal size and weight gain. This very preliminary data would hint that vagotomy attenuates the effects of RYGB on weight loss, without a major effect on food intake. These conclusions are limited by the small numbers and high mortality in our RYGB-V group and require further study.

There were two major learning curves with development of this model. First, we quickly learned that peripheral blood-draws were largely unsuccessful, and found placement of tunneled catheters superior. During our initial experience, we removed the tunneled catheters on POD 14 after glucose homeostasis testing and replaced them under anesthesia closer to final testing and scheduled necropsy. However, following two intra-operative arrests with catheter placements, including one in a sham surgery pig, we modified our protocol to allow tunneled catheters to remain until necropsy or catheter malfunction. Second, we learned that the thick stomach of the Ossabaw was not suitable for standard GIA staplers, and hand-sewn anastomoses became our practice.

Our model is limited by a relatively short, 60-day postoperative course, which restricts our ability to draw conclusions beyond the study time period or see complications that may develop beyond this time window. Furthermore, to date we have only studied a small number of Ossabaw in each subgroup, especially RYGB-V. Most of our deaths were early during the study period and part of our learning curve for operative technique. Lastly, while our preoperative weight, glucose, and insulin values demonstrate increased insulin resistance in high-fat-fed Ossabaws vs. lean Ossabaws, these values do not correlate perfectly with human values, and further work is needed to understand how the postoperative changes in GI physiology and glucose homeostasis relate to human counterparts.

## 5. Conclusion

In conclusion, the Ossabaw survival model for metabolic surgery appears to be appropriate, reproducible and should offer a novel way to evaluate the mechanism of effect of these procedures. This model is being used to evaluate the mechanism of T2DM resolution after bariatric surgery, the physiologic impact of novel surgical manipulations of the GI tract, and identification of novel targets for surgical and medical interventions.

## Figures and Tables

**Table 1:** Obesogenic-diet Ossabaw survival and complications.

	All	Sham	GJ	GJD	RYGB	RYGB-V
**N** a	32	2	10 (9 to OR)	7	8	5 (4 to OR)
N completing protocol/ N having GI surgery	23/30	1/2	8/9	6/7	6/8	1/2
(%)	77%	50%	89%	86%	75%	50%
Mean Postoperative survival, days	56.5	68.5	59.8	57.8	61.2	34
**Death**	9					
**Intraoperative or** **immediate** **postoperative**	5	1	2		0	2
During catheter placement	3	1 cardiac arrest during catheter replacement, POD 75	1 cardiac arrest during initial catheter placement			
			1 respiratory arrest during catheter replacement, POD 57			
During GI surgery	1					1 cardiac arrest during initial surgery
During re-exploration	1					1 explored for leak POD7, died of intra-operative hemorrhage
**Death prior to** **completion of** **study period**	4			1 died POD 5, pancreatitis	1 euthanized for eviceration on POD 35	1 euthanized for prolonged difficult recovery and decreased PO intake on POD11
					1 died POD 63, presumed C. diff	

a Pigs expiring of cardiac arrest during initial catheter placement (n = 2) not counted beyond first row as they did not undergo GI surgery.
